# Atrial approaches in mitral valve surgery: a propensity analysis of differences in the incidence of clinically relevant adverse effects

**DOI:** 10.1186/s13019-022-02058-4

**Published:** 2022-12-29

**Authors:** Carlos E. Obando, Javier D. Garzón, Lina M. Ramirez, Andrea C. Castillo, Albert F. Guerrero, Tomás Chalela, Diana C. Sandoval, Manuel Giraldo-Grueso, Nestor F. Sandoval, Jaime Camacho, Juan P. Umaña

**Affiliations:** 1 Cardiac Surgery Department, Fundación Cardioinfantil-Institute of Cardiology, Rosario University, Calle 163 A # 13B-60, 111831 Bogotá, Colombia; 2 General Surgery Resident, Ochsner Medical Center, New Orleans, LA USA; 3 Cardiothoracic Surgery, UPMC Children Hospital of Pittsburgh, Pittsburgh, USA; 4 Cardiothoracic surgery, Hospital universitario mayor MEDERI, Bogotá, Colombia; 5 General surgery Resident, Universidad del Rosario, Bogotá, Colombia

**Keywords:** Mitral valve, Cardiac surgical procedures, Surgical approaches, Complications, Postoperative period

## Abstract

**Background:**

The lack of evidence on complications using mitral valve approaches leaves the choice of risk exposure to the surgeon’s preference, based on individual experience, speed, ease, and quality of exposure.

**Methods:**

The present study analysed patients undergoing mitral valve surgery using a superior transseptal approach or a left-atrial approach between 2006 and 2018. We included first-time elective mitral valve procedures, isolated, or combined, without a history of rhythm disturbances. We used propensity score matching based on 26 perioperative variables. The primary endpoint was the association between the superior transeptal approach and clinically significant adverse outcomes, including arrhythmias, need for a permanent pacemaker, cerebrovascular events, and mortality.

**Results:**

A total of 652 patients met the inclusion criteria; 391 received the left atrial approach, and 261 received the superior transseptal approach. After matching, 96 patients were compared with 69 patients, respectively. The distribution of the preoperative and perioperative variables was similar. There was no difference in the incidence of supraventricular tachyarrhythmias or the need for treatment. The incidence of nodal rhythm (*p* = 0.008) and length of stay in intensive care (*p* = 0.04) were higher in the superior transseptal group, but the need for permanent pacemaker implantation was the same. Likewise, there was no difference in the need for anticoagulation due to arrhythmia, the incidence of cerebrovascular events or mortality in the postoperative period or in the long-term follow-up.

**Conclusion:**

We did not find an association with permanent heart rhythm disorders or any other significant adverse clinical outcome. Therefore, the superior transeptal approach is useful and safe for mitral valve exposure.

## Background

Despite the fact that minimally invasive surgery is perhaps the first alternative for the surgical treatment of mitral valve disease [1], the open approach plays a fundamental role, especially given the need for concomitant coronary artery revascularization procedures and/or intervention in multiple valves. It is necessary to be familiar with the different atrial approaches used in open surgery and to know the potential adverse effects derived from their use. There are multiple surgical methods to expose the mitral valve (MV), but the most used are the left lateral atrial and transseptal approaches or their extended superior transseptal version [2–5].

The most used incision is through the left atrium behind the interatrial groove, which provides satisfactory exposure of the valve and subvalvular apparatus. However, there are circumstances that limit the versatility of this type of access, such as the presence of a small left atrium, a deep thorax or simply the need for a greater degree of tissue dissection in the context of reoperation [1, 6].

The superior transeptal (TS) approach offers optimal exposure of the MV complex, even in the presence of hostile anatomical conditions and the event of reoperations, and it limits the need to extend the release of pleuropericardial, mediastinal adhesions or both, facilitates exposure and theoretically reduces the risk of bleeding. This approach was associated with a variety of complications, especially postoperative heart rhythm disorders, but the evidence is contradictory [7–12].

The lack of evidence of complications using either approach leaves the choice of exposure to the surgeon’s preference, based on individual experience, speed, ease, and quality of exposure, and less need for dissection in reoperations.

The present study approaches the problem from a different perspective to establish the association with clinically significant outcomes that lead to the need for additional therapeutic interventions, such as chronic anticoagulation due to arrhythmia, use of antiarrhythmic medication and electrical cardioversion, implantation of devices for rhythm control or both. The establishment of causal relationships between the type of surgical approach used for MV exposure and the development of postoperative complications will provide objective and useful elements when planning the strategy for open MV surgery.

## Methods

### Patients

This study was a retrospective cohort analysis of adult patients (over 18 years of age) who underwent cardiac surgery for the first time for conventional open intervention of MV between January 2006 and July 2018 at Fundación Cardioinfantil – Instituto de Cardiología. MV exposure was performed using a left atrial (LA) or TS approach. The study included patients with MV stenosis (MVS) or insufficiency (MVI) of any aetiology with indication for MV replacement (MVR) or repair (MVr) as a single procedure or combined with other types of valve surgery at the aortic, tricuspid or both, with and without coronary revascularisation.

Patients who underwent other types of MV access, emergency interventions, history of cardiac arrhythmia, use of devices for rhythm control, resynchronisation therapy, implantable cardioverter defibrillator (ICD) or both were excluded from the present study.

We searched for potentially eligible patients by convenience sampling, extracting the information from the institutional electronic medical records and selecting the patients who fulfilled the criteria, according to the Consort flow diagram.

### Ethics statement

The Clinical Research Ethics Committee of our institution approved the study (Act number 11–2017) and decided that there was no need for consent.

### Surgical technique

Surgical procedures were performed by the Institution's group of nine cardiovascular surgeons throughout the study period. Cardiopulmonary bypass (CPB) was established with arterial cannulation in the ascending aorta and bicaval venous cannulation in all cases, with normothermia or mild hypothermia via active cooling. The cardioplegic solutions used for myocardial protection included HTK solution (custodiol), cristaloid (St. Thomas, Del Nido) or blood cardioplegia using St. Thomas solution in a 4:1 ratio and Del Nido in a 1:4 ratio, administered via an antegrade and/or retrograde route.

#### Left atrium approach

After CPB was established, we performed a vertical left atriotomy anterior to the right superior pulmonary vein and posterior to the interatrial sulcus. The incision was extended superiorly behind the superior vena cava and inferiorly into the oblique fissure. MV retractors were used to expose the left atrium. Left heart venting was achieved via the right superior pulmonary vein, left atrium, pulmonary artery, or aortic root. After completion of the procedure, the left atriotomy was closed using a single layer of nonabsorbable suture.

#### Superior transseptal approach

After CPB was established, we performed a vertical right atriotomy parallel to the atrioventricular sulcus. A vertical septal incision was made through the fossa ovalis, avoiding the coronary sinus and extending into the roof of the left atrium. MV retractors were used to expose the left atrium. Left heart venting was achieved via the right superior pulmonary vein, left atrium, pulmonary artery or aortic root. After completion of the procedure, the left atriotomy in the roof of the left atrium, the vertical incision in the interatrial septum and the right atriotomy were closed using a single layer of nonabsorbable suture.

### Echocardiographic and haemodynamic data

Echocardiographic data were obtained from our institutional database. All preoperative studies were performed by our echocardiography laboratory, which is accredited by the Intersocietal Accreditation Commission. The variables evaluated were left ventricle ejection fraction (LVEF), pulmonary artery systolic pressure (PASP), left atrial diameter (LAD), type and severity of mitral and other types of valve dysfunction. The presence of haemodynamically significant CAD identified in the preoperative cardiac catheterisation was recorded in the database. The variables were categorised to define groups of outcomes according to the severity of the diagnosis.

### Data and follow-up

Patient records were reviewed to obtain demographic data, prior medical history, and intraoperative variables, including type of approach, valve interventions, coronary artery bypass grafting (CABG), myocardial protection strategy, CPB and cross-clamp times. During their hospitalisation, all patients were monitored with continuous telemetry, and any alteration of the rhythm was recorded in the medical records. Thirty-day postoperative follow-up was included in our database. Long-term follow-up was performed via telephone interviews and outpatient clinic visits. Patients were evaluated for the appearance of atrial fibrillation (Afib), flutter, other supraventricular arrhythmias, bradyarrhythmias or blocks, the use of antiarrhythmics, the need for electrical cardioversion, implantation of permanent pacemaker (PPM), and ICU length of stay.

### Statistical analysis

All preoperative, perioperative and 30-day variables were recorded in our database, which follows the guidelines established by the Society of Thoracic Surgeons. Long-term follow-up variables were recorded by extracting data from institutional registries and telephone survey.

Continuous variables are presented as medians and interquartile range (IQR). Preoperative and postoperative data were compared using Mann–Whitney U test for continuous variables. Regarding categorical variables, these are expressed as absolute and relative values within each category, groups were compared using the chi-squared test or Fisher's exact test. Statistical significance was assumed at *p* < 0.05. Data processing was performed using the Statistical Package for the Social Sciences—SPSS version 25 software for Windows.

To control the selection bias of the sample, we performed propensity score matching (PSM) using the nearest neighbour method, according to the similarities in the standardised differences between a case comparing it with 2 controls (matching 1:2), ordered from highest to lowest, without replacement of the data and setting a reference calliper of 0.2 [13].

Variables included in the calculation of the propensity score were sex, age, LVEF, PASP, LAD, preoperative creatinine, preoperative haematocrit, MVS (absent, mild, moderate, severe), MVI (absent, mild, moderate, severe), aortic valve disease (absent, stenosis, insufficiency, double injury), CAD, 3 vessel disease and/or left main trunk compromise, TV disease (≥ moderate), Euroscore II risk (%), diabetes mellitus, hypertension, COPD, stroke, CKD, PAD, pre-surgical use of beta-blockers and statins, MV surgery (valvuloplasty, bioprosthesis or mechanical prosthesis), CABG, aortic valve replacement, and tricuspid valve surgery.

A secondary analysis was performed by splitting the data into two time frames, 2006 to 2011 and 2012 to 2016. The endpoint of this analysis was to check if the results were consistent over time.

The Kaplan–Meier method and log-rank test were used to estimate and compare the survival rates between the 2 matched groups.

## Results

**Fig. 1:**
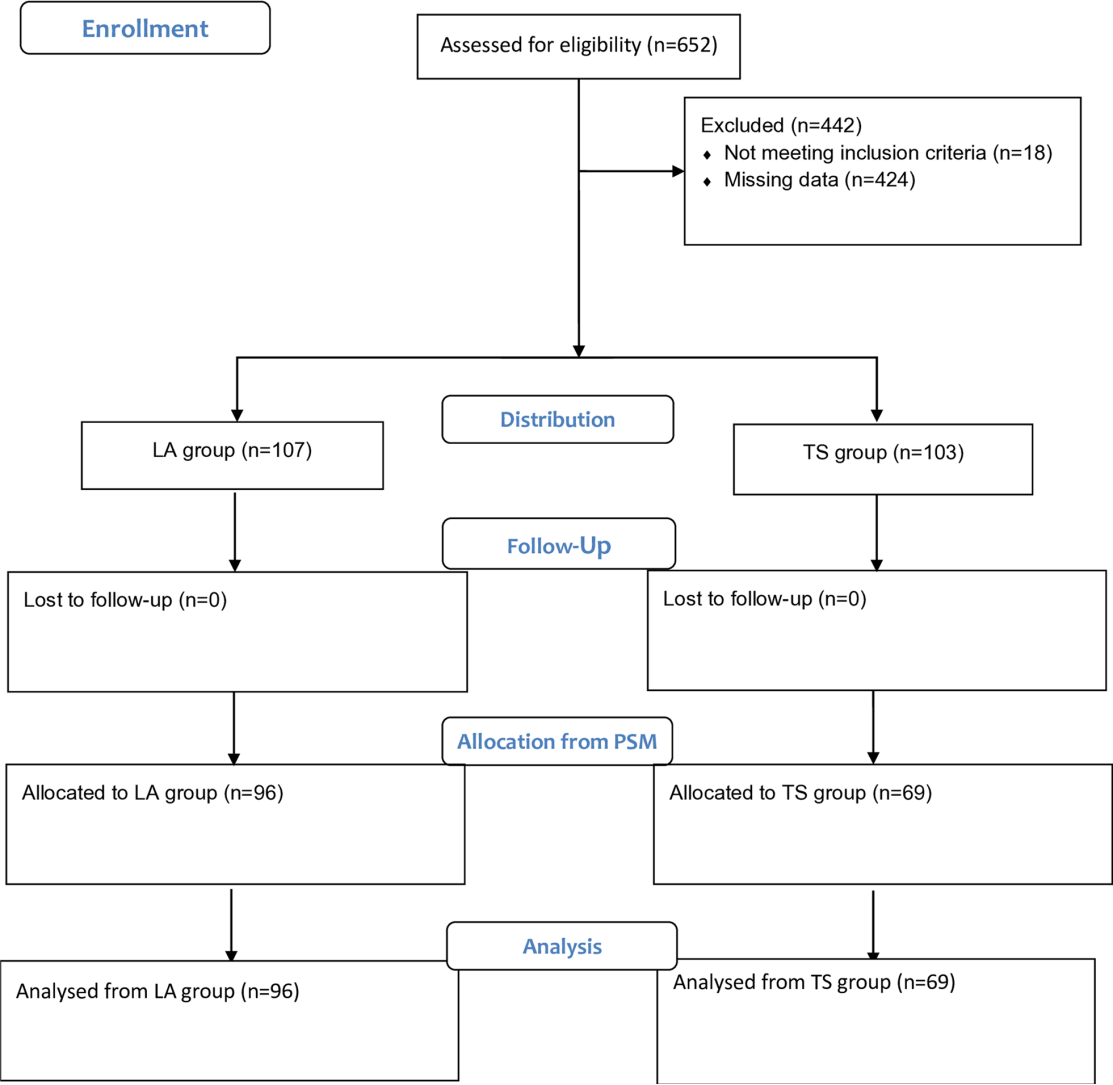
Consort 2010 flow diagram

Between January 2006 and July 2018, 652 patients who met the inclusion criteria underwent first-time isolated or combined MV surgery. Associated procedures included CABG, aortic valve replacement (AVR) and TV repair/replacement. A total of 391 patients received an LA approach (Group LA), and 261 patients received a TS approach (Group TS). We excluded 18 patients because of the type of surgical approach (minimally invasive) and 424 patients who had incomplete preoperative critical data, such as echocardiographic measures (Fig. 1).

**Fig. 2:**
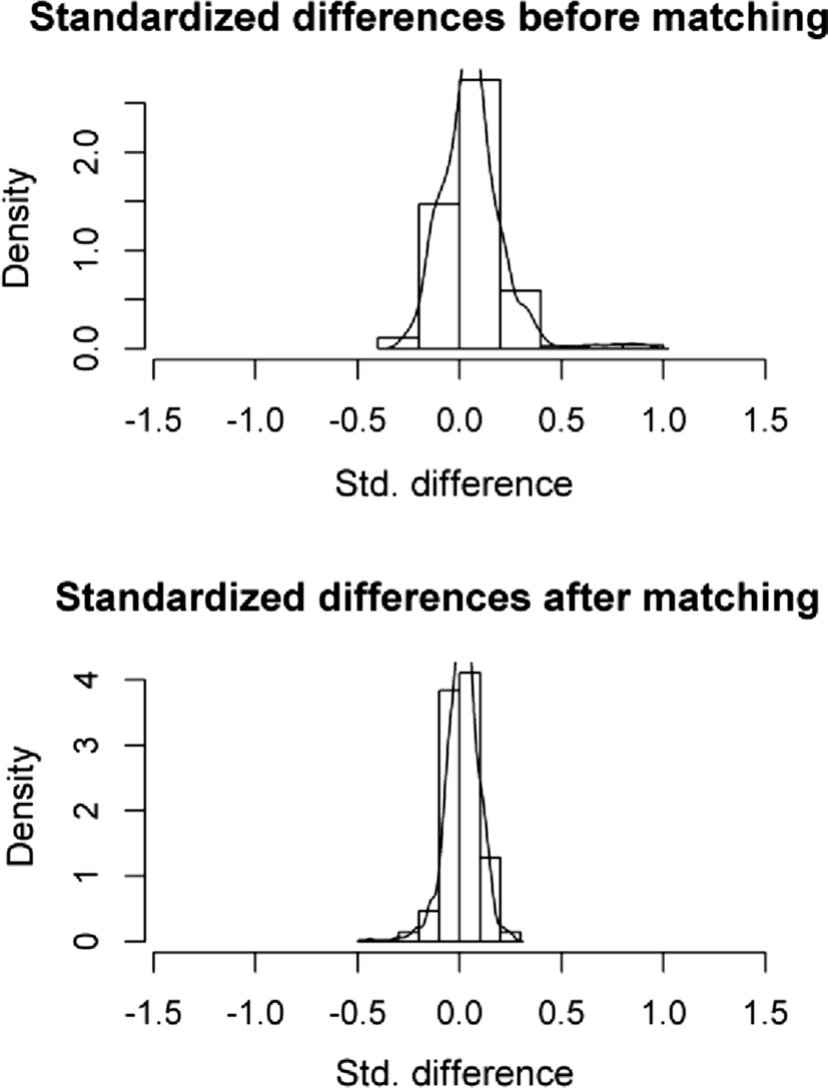
Standardised differences of the preoperative and perioperative variables before and after pairing by propensity scores

We obtained a new sample of 96 patients in the LA group and 69 patients in the TS group after PSM. Standardised differences were obtained, and an improvement in the sample heterogeneity was achieved because the post-PSM standardised differences were lower than 0.1 [13] (Fig. 2).

The preoperative and perioperative variables are illustrated in Table 1.

**Table 1:** Propensity score matching (PSM) of both groups for pre- and intraoperative variables

Pre-operative	Comparisons before matching				Comparisons after matching 1:2			
Variables	LA	TS	*p value*	SD	LA	TS	*p value*	SD
	*n* = 107	*n* = 103			*n* = 96	*n* = 69		
Sex (male)	65 (60.7)	67 (65)	*0.519*	0.09	59 (61.5)	41 (59.4)	*0.792*	− 0.045
Age (years)	62 (53–72)	65 (54–72)	*0.851*	− 0.016	64 (53–72)	64 (54–72)	*0.939*	0.001
LVEF (%)	41 (30–58)	50 (30–60)	*0.33*	0.119	42 (34–60)	50 (30–60)	*0.762*	0.01
PASP (mmHg)	38 (30–55)	50 (35–65)	***0.001***	0.421	40 (30–56)	43 (30–60)	*0.375*	0.027
Left atrial diameter (mm)	44 (39–50)	44 (39–48)	*0.946*	0.041	44 (39–50)	44 (38–48)	*0.962*	0.016
Creatinine (mg/dL)	1 (0.9–1)	1 (0.9–1)	*0.207*	− 0.042	1 (0.9–1)	1 (1–1.1)	*0.169*	0.005
PreOP haematocrit (%) *	41.1 (3.5)	40.9 (4.3)	*0.706*	− 0.056	40.9 (3.3)	41 (4.2)	*0.984*	0.042
Mitral stenosis			*0.632*				*0.83*	
Absent	92 (86)	89 (86.4)		–	83 (86.5)	60 (87)		–
Moderate	7 (6.5)	9 (8.7)		0.007	7 (7.3)	6 (8.7)		0
Severe	8 (7.5)	5 (4.9)		− 0.121	6 (6.3)	3 (4.3)		− 0.067
Mitral regurgitation			*0.608*				*1*	
Absent	2 (1.9)	4 (3.9)		–	2 (2.1)	2 (2.9)		–
Mild	8 (7.5)	11 (10.7)		0.103	8 (8.3)	6 (8.7)		0
Moderate	67 (62.6)	57 (55.3)		-0.146	60 (62.5)	42 (60.9)		0
Severe	30 (28)	31 (30.1)		0.045	26 (27.1)	19 (27.5)		0
Aortic valve disease			*0.89*				*0.924*	
Absent	63 (64.5)	61 (59.2)			61 (63.5)	41 (59.4)		
Stenosis	6 (5.6)	7 (6.8)		-	6 (6.3)	6 (8.7)		–
Insufficiency	23 (21.5)	25 (24.3)		0.064	21 (21.9)	16 (23.2)		0.034
Double injury	9 (8.4)	10 (9.7)		0.044	8 (8.3)	6 (8.7)		− 0.049
Tricuspid valve disease > moderate	43 (40.2)	51 (49.5)	*0.174*	0.186	39 (40.6)	31 (44.9)	*0.581*	0.014
Coronary disease	55 (51.4)	51 (49.5)	*0.785*	− 0.038	47 (49)	34 (49.3)	*0.968*	0.029
3 Vessel disease and left main trunk	19 (17.8)	28 (27.2)	*0.101*	0.211	19 (19.8)	16 (23.2)	*0.599*	0.032
Euroscore II risk (%)	3.5 (2.6–7.6)	4.3 (3.1–8.1)	*0.213*	− 0.087	3.5 (2.6–7)	4.2 (3.2–7.7)	*0.133*	0.043
Diabetes mellitus	26 (24.3)	25 (24.3)	*0.996*	− 0.001	23 (24)	16 (23.2)	*0.909*	0
Arterial hypertension	73 (68.2)	62 (60.2)	*0.225*	− 0.163	66 (68.8)	46 (66.7)	*0.777*	− 0.029
COPD	14 (13.1)	18 (17.5)	*0.376*	0.115	14 (14.6)	10 (14.5)	*0.987*	0
Stroke	5 (4.7)	7 (6.8)	*0.508*	0.084	5 (5.2)	5 (7.2)	*0.743*	0.057
CKD	13 (12.1)	11 (10.7)	*0.738*	− 0.047	13 (13.5)	9 (13)	*0.926*	0.047
PAD	3 (2.8)	3 (2.9)	*1*	0.006	3 (3.1)	3 (4.3)	*0.695*	0.043
Beta-blockers	65 (60.7)	55 (53.4)	*0.282*	− 0.147	57 (59.4)	38 (55.1)	*0.633*	− 0.043
Statins	62 (57.9)	52 (50.5)	*0.278*	− 0.148	54 (56.3)	37 (53.6)	*0.738*	− 0.014
Mitral intervention			*0.189*				*0.683*	
Mitral valve repair	56 (52.3)	41 (39.8)		–	49 (51)	31 (44.9)		–
Biological prosthesis	44 (41.1)	54 (52.4)		0.225	41 (42.7)	32 (46.4)		0.014
Mechanical prosthesis	7 (6.5)	8 (7.8)		0.046	6 (6.3)	6 (8.7)		0.054
CABG	53 (49.5)	51 (49.5)	*0.998*	0	46 (47.9)	33 (47.8)	*0.991*	0.014
Aortic valve replacement	22 (20.6)	32 (31.1)	*0.082*	0.226	21 (21.9)	19 (27.5)	*0.403*	0.078
Tricuspid intervention	8 (7.5)	21 (20.4)	***0.007***	0.319	7 (7.3)	6 (8.7)	*0.741*	− 0.036
Cardioplegia			*0.535*				*0.726*	
St Thomas Solution	32 (29.9)	25 (24.3)			29 (30.2)	15 (21.7)		
Del Nido	25 (23.4)	22 (21.4)		–	22 (22.9)	15 (21.7)		–
HTK Solution	15 (14)	13 (12.6)		–	12 (12.5)	10 (14.5)		–
Sanguineous	22 (20.6)	32 (31.1)		–	21 (21.9)	20 (29)		–
Others	13 (12.1)	11 (10.7)		–	12 (12.5)	9 (13)		-

*Mean (standard deviation), the statical significance is *p* < 0.05, shown in bolditalic and italic

Data are presented as frequencies and percentages (%) or as medians and interquartile range (IQR). unless otherwise specified

*PAD* peripheric arterial disease

*Standardised difference (SD)* it is the difference in the means divided by the standard error; an excellent balance between groups was defined as an absolute value less than 0.1 and up to 0.25 (corresponding to a small effect size)

Before matching, no statistically significant differences were found, except for PASP (*p* = 0.001) and tricuspid intervention (*p* = 0.007). No differences were observed in the distributions of other variables, such as the type and severity of MV dysfunction, aortic valve disease, comorbidity profile and intraoperative characteristics, such as type of mitral intervention, type of cardioplegia solution and cross-clamp and CPB time. Of the patients who underwent simultaneous AVR, only 0.95% received a mechanical prosthesis, with no differences in distribution between groups. All tricuspid valves were repaired.

The primary analysis according to PSM yielded 69 patients in the TS group and 96 in the LA group, with no significant differences between cohorts, except for a longer ICU stay (*p* = 0.002) and an increased prevalence of nodal rhythm in TS patients (*p* = 0.008). Table 2.

**Table 2:** Postoperative and post-discharge outcomes after propensity score matching

Post-operative	LA	TS	*p value*
*Variables after PSM*	*n* = 96	*n* = 69	
Cross-clamp time (minutes)	108 (74–130)	109 (70–141)	*0.38*
CPB time (minutes)	128 (98–156)	142 (100–170)	*0.14*
ICU stay (days)	3 (1–6)	4 (2–10)	***0.002***
Atrial fibrillation	33 (34.4)	25 (36.2)	*0.805*
Atrial flutter	5 (5.2)	4 (5.8)	*1*
Nodal rhythm	10 (10.4)	18 (26.1)	***0.008***
AV block	5 (5.2)	7 (10.1)	*0.228*
Sick sinus syndrome	1 (1)	0 (0)	*1*
Other SV arrhythmias	5 (5.2)	7 (10.1)	*0.228*
Antiarrhythmic medication	27 (28.1)	19 (27.5)	*0.934*
Electrical cardioversion	5 (5.2)	4 (5.8)	*1*
Device placement	7 (7.3)	7 (10.1)	*0.516*
Blood transfusion	54 (56.3)	48 (69.6)	*0.082*
Creatinine (mg/dL)	1.3 (1.1–1.6)	1.3 (1–1.6)	*0.543*
Oral anticoagulation	28 (29.2)	15 (21.7)	*0.284*
Perioperative mortality	4 (4.2)	7 (10.1)	*0.204*
*Post-discharge*			
SV arrhythmia	6 (6.2)	3 (4.3)	*0.595*
Device placement	5 (5.2)	3 (4.3)	*0.799*
Stroke	2 (2.1)	1 (1.4)	*1*
Valve reintervention	3 (3.1)	4 (5.8)	*0.366*
Chronic oral anticoagulation	29 (30.2)	16 (23.2)	*0.318*
Overall mortality	8 (8.3)	10 (14.5)	*0.211*

Data are presented as frequencies and percentages (%) or as medians and interquartile range (IQR). unless otherwise specified, the statical significance is *p* < 0.05, shown in bolditalic and italic

*AV Block* advanced atrioventricular block

**Fig. 3:**
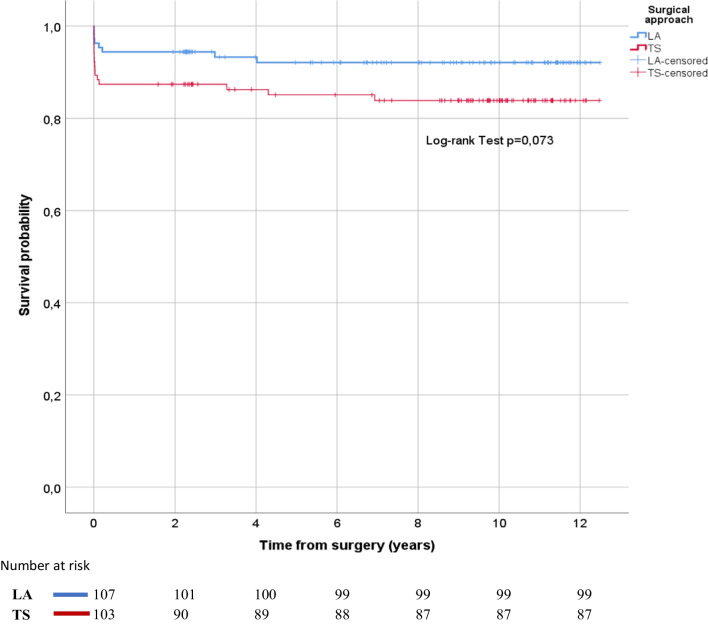
Kaplan–Meier survival curves

There were 24 deaths during the entire follow-up time (mean time from surgery 11.6 years for the LA group and 10.6 years for the TS group), but no significant differences were found between the type of surgical approach in the survival analysis (log-rank test *p* = 0.073) (Fig. 3).

The secondary analysis divided the sample according to the mean follow-up time into 2 groups. The first sub analysis included the date of surgery from January 2006 to July 2011, and the second subgroup was from July 2011 to December 2016. No significant differences were found between patients, except for a longer ICU stay (*p* = 0.04) and an increased prevalence of nodal (*p* = 0.023) and other SV arrhythmias (X^**2**^ *p* = 0.045) in TS patients for the first sub analysis group (Table 3).

**Table 3:** Postoperative and post-discharge outcomes between the years 2006 and 2011

Outcomes 2006–2011	LA	TS	*p value*
*Variables after PSM*	*n* = 60	*n* = 53	
Euroscore II risk (%)	3.5 (3.5–8)	4.7 (3.5–8.5)	*0.235*
Cross-clamp time (minutes)	106 (61–134)	100 (69–140)	*0.739*
CPB time (minutes)	123 (93–160)	135 (96–160)	*0.372*
ICU stay (days)	3 (1–6)	4 (2–10)	***0.04***
Atrial fibrillation	19 (31.7)	19 (35.8)	*0.639*
Atrial flutter	2 (3.3)	2 (3.8)	*1*
Nodal rhythm	6 (10)	14 (26.4)	***0.023***
AV block	1 (1.7)	4 (7.5)	*0.185*
Sick sinus syndrome	0 (0)	0 (0)	*–*
Other SV arrhythmias	0 (0)	4 (7.5)	***0.045***
Antiarrhythmic medication	17 (28.3)	14 (26.4)	*0.82*
Electrical cardioversion	2 (3.3)	4 (7.5)	*0.417*
Device placement	2 (3.3)	5 (9.4)	*0.25*
Blood transfusion	36 (60)	39 (73.6)	*0.127*
Creatinine (mg/dL)	1.3 (1.1–1.6)	1.3 (1–1.6)	*0.666*
Oral anticoagulation	15 (25)	9 (17)	*0.298*
Perioperative mortality	4 (6.7)	6 (11.3)	*0.511*
*Post-discharge*			
SV arrhythmia	1 (1.7)	3 (5.7)	*0.466*
Device placement	1 (1.7)	3 (5.7)	*0.466*
Stroke	2 (3.3)	1 (1.9)	*1*
Valve reintervention	3 (5)	4 (7.5)	*0.597*
Chronic oral anticoagulation	15 (25)	10 (18.9)	*0.433*
Overall mortality	6 (10)	9 (17)	*0.275*

Data are presented as frequencies and percentages (%) or as medians and interquartile range (IQR). unless otherwise specified, the statical significance is *p* < 0.05, shown in bolditalic and italic

*AV Block* advanced atrioventricular block

For the second sub analysis group, no significant differences were found between patients, except for longer ICU stay (*p* = 0.04) and an increase cross-clamp (*p* = 0.024) and CPB time (*p* = 0.049) in TS patients (Table 4).

**Table 4:** Postoperative and post-discharge outcomes between the years 2012 and 2016

Outcomes 2012—2016	LA	TS	*p value*
*Variables after PSM*	*n* = 36	*n* = 16	
Euroscore II risk (%)	2.9 (2.2–5.1)	3 (2–4.4)	*0.874*
Cross-clamp time (minutes)	110 (87–130)	133 (105–149)	***0.024***
CPB time (minutes)	130 (108–154)	155 (129–172)	***0.049***
ICU stay (days)	3 (1–6)	7 (4–13)	***0.007***
Atrial fibrillation	14 (38.9)	6 (37.5)	*0.924*
Atrial flutter	3 (8.3)	2 (12.5)	*0.637*
Nodal rhythm	4 (11.1)	4 (25)	*0.231*
AV block	4 (11.1)	3 (18.8)	*0.662*
Sick sinus syndrome	1 (2.8)	0 (0)	*1*
Other SV arrhythmias	5 (13.9)	3 (18.8)	*0.689*
Antiarrhythmic medication	10 (27.8)	5 (31.3)	*1*
Electrical cardioversion	3 (8.3)	0 (0)	*0.544*
Device placement	5 (13.9)	7 (10.1)	*1*
Blood transfusion	18 (50)	9 (56.3)	*0.677*
Creatinine (mg/dL)	1.3 (1.2–1.6)	1.3 (1.1–1.6)	*0.959*
Oral anticoagulation	13 (36.1)	6 (37.5)	*0.924*
Perioperative mortality	0 (0)	1 (6.3)	*0.308*
*Post-discharge*			
SV arrhythmia	5 (13.9)	0 (0)	*0.308*
Device placement	4 (11.1)	0 (0)	*0.299*
Stroke	0 (0)	0 (0)	*-*
Valve reintervention	0 (0)	0 (0)	*-*
Chronic oral anticoagulation	14 (38.9)	6 (37.5)	*0.924*
Overall mortality	2 (5.6)	1 (6.3)	*1*

Data are presented as frequencies and percentages (%) or as medians and interquartile range (IQR). unless otherwise specified, the statical significance is *p* < 0.05, shown in bolditalic and italic

*AV Block* advanced atrioventricular block

No significant differences in the perioperative or overall mortality were found in either group. However, a noticeable difference was observed in the mortality figures in each of the intervention groups between the two periods.

There were significant differences in the myocardial protection strategies used in each time. During the first time, HTK solution (Custodiol) was used in 19.5% of interventions and blood cardioplegia with Del Nido solution was used in 27.4%. During the second, they were used in 0% and 11.5% respectively. Likewise, during the first period there was a tendency to use retrograde cardioplegia more frequently as a complementary route of administration.

## Discussion

Since the first description of the TS approach by Guiraudon and colleagues in 1991 [4, 8], there has been controversy on its relationship with postoperative heart rhythm disorders, the need for PPM, and postoperative bleeding. Available evidence primarily comes from retrospective studies and a few randomised prospective studies without adequate power, which explains why it is contradictory and not widely applicable [14].

Our study addressed this problem using a cohort model in which the differences in prognosis of MV surgery were analysed after the use of the left atrial vs. superior transseptal approach. Because there was a non-random distribution between groups, conditions that could influence the selection of the technique, such as reoperations or emergency surgeries, were excluded from the analysis. Patients with a history of arrhythmia, chronic anticoagulation or PPM were also excluded because the objective of this study was to precisely elucidate the effects of the use of the two main mitral approach strategies in the development of rhythm disturbances, the need for PPM, and the use of postoperative anticoagulation and antiarrhythmic medication. We used PSM to further minimise biases inherent to retrospective analyses.

After matching with propensity scores, no significant differences were observed in the postoperative incidence of Afib/flutter or other types of supraventricular arrhythmias between groups. There was a higher incidence of nodal rhythm (*p* = 0.008) and length of stay in the ICU (*p* = 0.04) in patients undergoing a TS approach. However, these rhythm disorders were mostly transitory. Therefore, they did not result in a significant difference in the need for PPM implantation. Similar findings were reported in other studies and reflect the benign behaviour of early rhythm disorders related to the TS approach [15, 16]. Because we found no differences in the incidence of Afib/flutter and the use of anticoagulation and antiarrhythmic medications, the need for postoperative electrical cardioversion was the same between groups. Our results are consistent with the observations of the prospective randomised study by Gaudino et al., who did not identify significant differences in the incidence of cardiac rhythm disturbances in patients whose preoperative rhythm was a normal sinus rhythm [17]. In contrast, Rezahosseini et al., in a retrospective cohort analysis performed via pairing with propensity scores that gathered 815 patients, observed a significant increase in the prevalence of postoperative Afib in patients who received the TS mitral approach (36.8% vs. 27.5%, *p* = 0.019), with no differences in the need for a perioperative temporary pacemaker between the groups. Although it was essentially a transient dysfunction, our higher early incidence of nodal rhythm partially contributed to the longer stay in the ICU with the TS approach. However, it is clear that the definition of this outcome was due to a multifactorial origin. Turkyilmaz and Kavala, in a retrospective analysis using propensity scores, identified a significant increase in ICU stay (*p* < 0.001) and hospitalisation (*p* < 0.001) associated with the TS approach despite a lack of significant differences in the prevalence of postoperative rhythm disturbances. They instead identified perioperative bleeding as the main factor influencing this outcome (*p* < 0.001) [16].

The rhythm disturbances correlated with the TS approach may be explained because of the proximity of the sinus node artery, which is easily injured and leads to ischaemia and resultant nodal dysfunction. The incision also causes internodal pathway disruption, and scar formation may block impulses from the sinus node [18].

Nienabber et al., in a retrospective analysis of 531 patients comparing the LA approach with the so-called mini-transseptal access, limited to the interatrial septum without extension to the atrial roof, observed a significant increase in the incidence of junctional rhythm (8.7% vs. 4.2%, *p* = 0.035) and the need for PPM (10.5% vs. 5.1%, *p* = 0.025). However, multivariate analysis showed that TS access was not an independent predictor for the development of rhythm alterations or the need for PPM, and the latter is specifically related to the presence of redo sternotomy [19]. Lukac et al. also identified a greater need for PPM in their retrospective cohort analysis of 577 patients (*p* = 0.010) undergoing the TS approach, which was primarily related to a higher incidence of sinus node dysfunction (*p* = 0.017) [20]. In the long-term follow-up of our cohorts, clinical stability was evidenced without significant differences in the incidence of arrhythmias, the need for antiarrhythmic medication, the use of oral anticoagulation in non-carriers of mechanical valves, or the incidence of cerebrovascular events. The need for late PPM implantation was also similar between groups.

We did not observe a significant difference in perioperative (*p* = 0.204) or late (*p* = 0.211) mortality associated with the use of a TS approach. Gaudino et al. [17] and Aydin et al. [15] specifically evaluated the outcome of mortality without being able to establish a relationship with the type of atrial approach. No evidence is available from prospective studies showing an association between the use of the ST approach and an increase in mortality [14]. The recent meta-analysis by Harky et al. compared the outcomes in MV surgery of these two types of approaches, but it included limited transseptal access and superior transseptal access in the TS group. A total of 4537 patients were included and evaluated for primary outcomes, operative mortality and PPM implantation. The mortality outcome was similar between the groups, unlike the need for PPM implantation and the incidence of new-onset AF, which were higher in the TS group. Analysis of the isolated MV surgery subgroup did not show any significant difference. Unlike our study, the distribution of other concomitant valve procedures was not symmetrical, which could influence the higher incidence of postoperative rhythm disorders and the need for PPM [21].

The high mortality rate observed in both intervention groups is striking. However, both isolated mitral procedures and procedures combined with valve interventions in other locations and/or with coronary revascularisation were included in the present analysis. The secondary analysis in different periods showed that these high mortality values were primarily conditioned by the results obtained in the initial period. Important factors that may have influenced the improvement of the postoperative prognosis likely include improvements in surgical technique and anaesthesia and advances in cardiopulmonary bypass technology and intensive care management.

This is a retrospective study based on observational data and is therefore limited by the biases inherent in this type of analysis. Strict criteria were used for the inclusion of patients in the final propensity analysis obtaining two completely comparable populations with a homogeneous distribution of variables, however this significantly reduced the sample size. More studies are needed in this subject.

## Conclusions

This study did not find significant differences in the postoperative incidence of permanent heart rhythm disorders or any other clinically significant adverse outcome in relation to the type of atrial approach. Therefore, the superior trans septal approach represents an useful and safe alternative for mitral valve exposure.

## Data Availability

The database collected in the study is available from the corresponding author on reasonable request.

## Abbreviations

Afib: Atrial fibrillation
AV: Atrioventricular
AVR: Aortic valve replacement
CABG: Coronary artery bypass grafting
CAD: Coronary artery disease
COPD: Chronic obstructive pulmonary disease
CKD: Chronic kidney disease
CPB: Cardiopulmonary bypass
EF: Ejection fraction
LA: Left atrial
ICD: Implantable cardioverter defibrillator
ICU: Intensive care unit
LAD: Left atrial diameter
LVEF: Left ventricle ejection fraction
MV: Mitral valve
MVI: Mitral valve insufficiency
MVr: Mitral valve repair
MVR: Mitral valve replacement
MVS: Mitral valve stenosis
PASP: Pulmonary artery systolic pressure
PPM: Permanent pacemaker
PSM: Propensity score matching
SV: Supraventricular
TS: Superior transseptal approach
TV: Tricuspid valve
